# The Practitioners' Bookshelf

**Published:** 1892-11-26

**Authors:** 


### THE PRACTITIONERS' BOOKSHELF.

### MEDICAL MICROSCOPY.*

The author states that in this work an effort has been made to lay before practitioners and students the most simple methods of preparing microscopic sections, and of the examination of urinary deposits, sputa, blood, &c; while, at the same time, for those who wish to extend their studies further, more elaborate methods have been detailed, and chapters added on the examination of food and bacteriological methods. From a joint author of the excellent "Clinical and Practical Pathology" we should expect to find the subject of this more recent work treated in a clear and practical style, and in this we are in no way disappointed. It is evidently written by one who has a very thorough practical knowledge of his subject, and consequently will be of great value to senior students and practitioners too, who desire to be up to date in their methods of histological investigation as an aid to diagnosis. It is well printed in clear type, and is a very handy volume. The numerous illustrations, a by no means unimportant feature in a work of this character, are well chosen, and will be most helpful. We congratulate the author on the result of his labours, and heartily commend the book as one of the best manuals on clinical histology.

### ARTIFICIAL FEEDING AND FOOD DISORDERS OF CHILDREN.†

"Children are generally overdosed with cod liver oil, syrup of phosphates, and chemical food." This is the burden of Dr. Cheadle's valuable book, not the dosing, but the dietary of infants; not the cure, but the prevention of infantile diseases; not the attempted repair by subsequent overfeeding of an enfeebled constitution, but the building up of a strong constitution by proper and intelligent feeding in infancy. Dr. Cheadle's lectures were addressed to medical men and students, but they are so lucid and direct that all who have any knowledge of physiology and dietetics can understand them. We warmly recommend the volume to those who have the care of children, and who think that the insurance of the health of their little charges is worth the taking the trouble of mastering the scientific principles on which their feeding should be based. Dr. Cheadle first shows the proportions in which the albuminates, the fats, and carbo-hydrates exist in human milk, and then describes how cow's milk can be treated to imitate these proportions. He strongly urges that all milk used in the nursery should be boiled, in order that fermentative changes should be effectually stopped, and also that the germs of infectious diseases, such as scarlet fever, typhoid, and tuberculosis, should be destroyed; and he says, "I have observed generally that those households where all milk is boiled enjoy a singular freedom from infectious diseases." The advantages of adding lime water, barley water, bicarbonate of soda, and cream, are discussed, and exact instructions given; also the value of peptonised milk, condensed milk, and artificial human milk. The conditions under which other artificial foods must be used are then described, and the preparation and uses of bread jelly, and raw meat juice are clearly given. Dr. Cheadle points out how starch foods, such as arrowroot and corn-flour, are inadmissible for infants, who have not the power of digesting starch till they are six months old; and that even the much-vaunted malted foods—in which the starch is changed into dextrine—are absolutely unsatisfactory unless taken with milk. The colic, flatulence, and diarrhœa of bottle-fed children are due to incorrect feeding, and when a child is seen to suffer or to do badly on one artificial food or one kind of milk, it is not enough to suddenly put it on another, but to study the causes of failure and to ascertain by a correct knowledge of food values the element deficient in the infant's diet. "The occurrence of rickets," says Dr. Cheadle, "is too often a grave reflection upon medical man, or nurse, or mother, under whose directions the diet of the child has been regulated." Want of knowledge can nowadays be no excuse for the infliction of suffering, and an opportunity to know how to diet infants and to ensure their health, is given by Dr. Cheadle's admirable book, and should be taken advantage of by all intelligent persons having the care of infants.

* "Medical Microscopy." By Frank Wethered, M.D.Lond. Illustrated; Pp. 400. (Lewis, London.)

† "Artificial Feeding and Food Disorders of Children." By W. B. Cheadle, M.A., M.D. Cantab, F.R.C.P. (Smith, Elder, and Co., London, 1892).

